# Oxidative Stress-Associated Male Infertility: Current Diagnostic and Therapeutic Approaches

**DOI:** 10.3390/medicina60061008

**Published:** 2024-06-20

**Authors:** Haritha Pavuluri, Zahra Bakhtiary, Manesh Kumar Panner Selvam, Wayne J. G. Hellstrom

**Affiliations:** Department of Urology, Tulane University School of Medicine, New Orleans, LA 70112, USA; hpavuluri@tulane.edu (H.P.); zbakhtiary@tulane.edu (Z.B.); mpannerselvam@tulane.edu (M.K.P.S.)

**Keywords:** oxidative stress, male infertility, reactive oxygen species, antioxidants

## Abstract

Infertility is a prevalent global issue affecting approximately 17.5% of adults, with sole male factor contributing to 20–30% of cases. Oxidative stress (OS) is a critical factor in male infertility, disrupting the balance between reactive oxygen species (ROS) and antioxidants. This imbalance detrimentally affects sperm function and viability, ultimately impairing fertility. OS also triggers molecular changes in sperm, including DNA damage, lipid peroxidation, and alterations in protein expression, further compromising sperm functionality and potential fertilization. Diagnostic tools discussed in this review offer insights into OS markers, antioxidant levels, and intracellular ROS concentrations. By accurately assessing these parameters, clinicians can diagnose male infertility more effectively and thus tailor treatment plans to individual patients. Additionally, this review explores various treatment options for males with OS-associated infertility, such as empirical drugs, antioxidants, nanoantioxidants, and lifestyle modifications. By addressing the root causes of male infertility and implementing targeted interventions, clinicians can optimize treatment outcomes and enhance the chances of conception for couples struggling with infertility.

## 1. Introduction

Infertility is defined by the World Health Organization (WHO) as the inability to conceive after 12 months or more of regular unprotected sexual intercourse [1]. Approximately, 17.5% of the adult population experience infertility (approximately 1 in 6), with a lifetime prevalence of 17.8% in high-income countries and 16.5% in low- and middle-income countries [1]. Studies estimate that 20–30% of infertility cases are solely due to male factor, with the male contributing to approximately 50% of infertility in couples [2]. The prevalence of infertility among couples globally appears to be highest in South Asia, sub-Saharan Africa, North Africa, the Middle East, Central and Eastern Europe, and Central Asia [3]. However, the global prevalence of male infertility is poorly documented, particularly in developing countries, and thus remains mostly unknown [4]. It is of significance to differentiate between primary infertility (difficulty or inability to achieve a pregnancy with no prior pregnancy) and secondary infertility (difficulty achieving an additional pregnancy after having at least one delivery). Secondary infertility is five times more prevalent than primary infertility [3,4]. This is especially applicable to low- and middle-income countries, possibly due to the increased incidence of post-infection infertility in these countries [4]. 

In a clinical environment, male infertility is often treated secondary to idiopathic hypogonadotropic hypogonadism (IHH), with less emphasis on molecular contributors. Treatments include human chorionic gonadotropin, follicle-stimulating hormone, and gonadotropin-releasing hormone. Semen analysis is a common diagnosis modality utilized to evaluate sperm parameters. Diminished semen quality has been demonstrated to be a major predictor of male fertility outcome and the likelihood of pregnancy [5]. Per some studies that retroactively evaluated semen records, semen quality has decreased over several decades [6,7,8]. Several factors may be contributing to this rise in male factor infertility, such as pesticides, heavy metals, industrial chemicals, obesity, tobacco and alcohol use, poor nutrition, sedentary lifestyle, physiological issues, and genetic factors [9]. In addition, pathologic causes that lead to male infertility can be subcategorized as testicular, pretesticular, extratesticular, and idiopathic infertility [10]. Idiopathic male infertility comprises 30–40% of cases [11]. The primary common pathway leading to idiopathic male infertility involves numerous endogenous and exogenous factors that induce oxidative stress (OS) [12,13]. Increased production of reactive oxygen species (ROS) damages the sperm membrane and DNA, a phenomenon observed in 30–80% of infertile males [14].

While there is significant literature on the various positive and negative contributors to male infertility, including the role of ROS, there is a lack of comprehensive review literature evaluating the various diagnostic modalities for ROS-induced infertility while also detailing both medical and lifestyle therapeutic modalities.

## 2. OS-Associated Male Infertility

**Figure 1:**
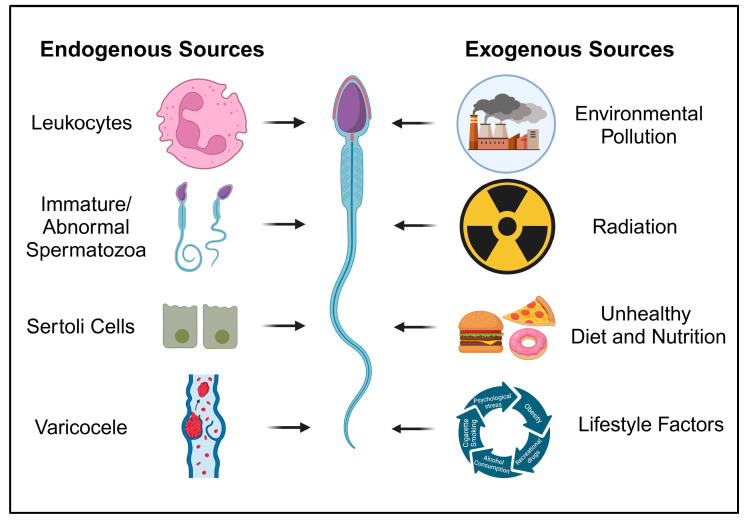
Exogenous and endogenous sources involved in generation of ROS leading to sperm dysfunction.

Physiological levels of ROS are required to activate transcription factors involved in the intracellular signaling that mediates essential physiological processes like spermatogenesis, sperm maturation, capacitation, hyperactivation, chemotaxis, acrosome reaction, and sperm–oocyte fusion [15,16]. High ROS production can result in oxidative damage to proteins, lipids, and nucleic acids, while antioxidant molecules work to maintain a redox balance and prevent biological system damage [17,18]. Excessive ROS production leads to sperm injury because spermatozoa are highly susceptible to oxidation. This susceptibility arises from a substantial presence of unsaturated fatty acids in the membrane and a deficiency in cytoplasmic antioxidant enzymes [17]. Various significant exogenous factors may promote redox alterations, thereby contributing to male infertility (Figure 1). These factors include environmental pollution, bacterial/viral infections, and lifestyle factors such as obesity, tobacco and alcohol abuse, the presence of varicocele, and sexually transmitted diseases/disorders [19,20].

Male infertility can be influenced by various endogenous sources of ROS in seminal plasma. One significant contributor is leukocytes, particularly polymorphonuclear leukocytes and macrophages, originating from the seminal vesicles and prostate gland. In the presence of urogenital infections or inflammation, these leukocytes exhibit a heightened immune response, generating up to 100 times more ROS and thus leading to OS [21]. Immature spermatozoa, with excess residual cytoplasm (ERC) due to disrupted spermiogenesis, are another major source of seminal ROS. The retained cytoplasm contains metabolic enzymes, such as glucose-6-phosphate dehydrogenase and nicotinamide adenine dinucleotide phosphate (NADPH) oxidase, contributing to ROS production. Sertoli cells, involved in spermatogenesis, have been documented to generate ROS, with scavestrogens inhibiting their production [22]. Additionally, varicocele, an abnormal venous dilation of the spermatic cord in up to 30% of infertile men, induces testicular hyperthermia and hypoxia, resulting in OS-induced sperm dysfunctions. Semen samples from varicocele-affected infertile patients show elevated ROS levels, correlating with the grade of varicocele. These endogenous sources collectively highlight the complex interplay of cellular components and physiological conditions contributing to ROS-mediated male infertility [21,22,23,24,25].

Elevated ROS levels are postulated to inhibit the motility of spermatozoa and interfere with their overall ability to attach to the oocyte [26]. Higher blood leukocytes, ROS production, increased plasma lipid peroxidation (LPO), and reduced total antioxidant capacity (TAC) were noted in oligoasthenozoospermic (OAT) men compared to the sperm of healthy fertile men [27]. These factors are implicated in cellular dysfunction, affecting the total number, motility, and concentration of spermatozoa [27]. Specifically, LPO products interact with amino residues, leading to protein oxidation and thus affecting the structural and functional aspects of proteins in sperm, particularly in the sperm mitochondrial electron transport chain [28]. Furthermore, LPO products can result in loss of membrane integrity and alterations in motility, thus negatively impacting interactions between sperm and oocytes [29]. Due to the deficiency of adequate antioxidant systems and a lack of complete DNA repair pathways, spermatozoa are highly susceptible to DNA oxidation [28]. 

ROS are generated across cellular metabolism, particularly during the production of adenosine 5 triphosphate (ATP) by mitochondria in leukocytes and immature spermatozoa; this occurs through electron transport or some enzymatic activities in seminal plasma [30,31,32]. The amount of ROS may be affected by factors such as highly activated leukocytes due to infection or inflammation [33] as well as exposure to radiation or toxins [31,34]. The overproduction of ROS disrupts the balance between ROS and the body’s natural antioxidant mechanism, consequently impairing spermatozoa function; this imbalance leads to the peroxidation of cell membrane lipids, which contain high amounts of polyunsaturated fatty acids (PUFAs) [35]. The outflow of ATP from the inner cell and reduced viability contribute to sperm morphological defects and motility dysfunction, ultimately diminishing sperm quality and fertility [36,37]. Studies have demonstrated that DNA damage, which cannot be repaired due to the absence of a cytoplasmic enzyme repair system [28,31,37], along with poor chromatin packaging, enhances the induction of apoptotic pathways [37]. It has also been demonstrated that a decrease in antioxidant levels may initiate excessive ROS generation [38]. For example, deficiencies in vitamin C or zinc result in sperm DNA fragmentation and male factor infertility [39,40,41].

### 2.1. Leukocytospermia and OS 

Leukocytospermia is characterized by the presence of more than 1 × 10^6^/milliliter (mL) white blood cells (WBCs) in semen and is often linked with unexplained male infertility [42]; these cells are responsible for generating ROS [17]. The main sources of leukocytes originate from the prostate, seminal vesicles, vas deferens, and epididymis, with minimal contribution from the testis due to the presence of the blood–testis barrier [43]. While leukocytospermia may suggest an infectious process such as male accessory gland infection, recent studies indicate that elevated WBC levels can occur without infection or immune response [44]. Moreover, leukocytospermia has been linked to detrimental effects on sperm function attributed to ROS generation [44,45,46]. Increased seminal WBC levels were observed in infertile men compared to healthy controls. Notably, leukocytospermia showed a significant correlation with reduced sperm count, motility, and altered morphology due to the production of ROS when compared to non-leukocytospermic patients. Additionally, even at low concentrations, semen WBCs were positively correlated with OS [17]. 

Research by Sharma and colleagues revealed increased ROS levels even with leukocytospermia levels below 0.2 × 10^6^/mL, suggesting that leukocyte levels below the WHO cut-off value may still be harmful [47]. Athayde et al. demonstrated that men with a normal Endtz test had a 24% chance of natural conception, while men with leukocytospermia levels below 1 × 10^6^/mL experienced a 16% decrease in natural conception rates during a 12-month follow-up [48]. Increased OS observed during leukocytospermia is believed to result from a defective ROS scavenging system [49]. Hence, it is evident that ROS can contribute to male factor infertility through various exogenous factors. 

Among various leukocyte subtypes, peroxidase-positive cells, specifically neutrophils and macrophages, emerge as the primary sources of ROS production [50]. In ejaculates obtained through electroejaculation, these two leukocyte subpopulations, identified through immunohistochemical staining, were the major contributors to leukocytospermia in men with spinal cord injury [51,52]. Lymphocytes, particularly activated T cells, were also significant contributors to leukocytospermia in men with spinal cord injury, as indicated by immunophenotypic analysis using flow cytometry. Notably, many of these T cells co-express the human leukocyte antigen HLA-DR and CD25. However, no significant B-cell population was observed [53]. 

In response to infection or inflammation, these cells initiate a defense mechanism by producing ROS at a rate 100 times higher than the normal production rate and also stimulate NADPH production through the hexose monophosphate (HMP) shunt pathway [54]. The generation of ROS is a key mechanism in the immune defense induced by leukocytes, as these cells are potent inducers of OS [55]. Moreover, the increase in proinflammatory mediators, particularly IL-6, IL-8, and TNFα, coupled with a decrease in antioxidants during inflammatory reactions, can trigger a respiratory burst leading to high OS [56]. 

### 2.2. OS and Molecular Alterations in Sperm

As previously mentioned, OS adversely affects the physiological function of spermatozoa [57,58], inducing damage to various biomolecules such as DNA, lipids, and proteins; this damage can lead to reproductive system disorders, including infertility [59,60,61,62]. Sperm are especially vulnerable to oxidative damage during spermiogenesis and storage in the epididymis, as they lack protection from antioxidant-rich seminal plasma [63]. Furthermore, OS in male germ cells is associated with damage to the nuclear and mitochondrial genome as well as the epigenome [64]. Telomeres, hexameric guanine-rich repeats, are highly susceptible to free-radical attack, leading to the generation of mutagenic 8-hydroxy-deoxyguanine adducts and DNA breaks [65,66,67]. Several studies on sperm and seminal plasma have focused on understanding the impact of OS on cellular pathways and sperm proteins associated with reproduction (Table 1 and Table 2).

Persistent OS in semen can alter sperm protein expression. Hamada et al. detected 1265 and 1343 sperm proteins in high and low levels of seminal ROS samples, respectively [68]. A liquid chromatography–tandem mass spectrometry analysis revealed 74 differentially expressed proteins (DEPs) in sperm due to OS [69]. These DEPs were mainly involved in the CREM signaling pathway regulating spermatid differentiation. Key proteins like HIST1H2BA and MDH2 were suggested as OS biomarkers [69]. Computational approaches also identified that ACE and HSPA2 are linked with altered sperm function in patients with high seminal ROS levels [76]. Another study identified a set of six proteins (CLGN, TPPII, DNAI2, EEA1, HSPA4L, and SERPINA5) common in patients with varying levels of seminal OS, which impact sperm’s physiological function [70]. Dias et al. observed alterations in sperm and seminal plasma proteomes of fertile men with high ROS levels, indicating the activation of proteasomal systems and antioxidant defense mechanisms [71].

Proteomic studies on the seminal plasma of infertile men with high seminal OS levels are limited. Sharma et al. compared seminal plasma proteomes of men with high versus low ROS levels and reported 14 DEPs [72]. Among these, ACPP and AZGP1 were regulated by the androgen receptor. MME and FAM3D proteins were proposed as biomarkers for evaluating fertility status in men with varying ROS levels [73]. A similar proteomic approach was used to identify 94 DEPs in the seminal plasma of normozoospermic men with high OS, suggesting mucin-5B as a marker of OS in semen [74]. Herwig et al. identified a set of 46 seminal plasma proteins in patients with idiopathic oligoasthenoteratozoospermia (iOAT), elucidating the etiology of OAT attributed to OS [75].

As described above, oxidative attacks secondary to ROS on the sperm membrane lipids initiate the LPO cascade [77]. While PUFAs provide fluidity and flexibility for membrane fusion events during fertilization, their double bonds make them vulnerable to free radical attacks, initiating the LPO cascade. This cascade leads to a loss in membrane integrity, impaired cell functions, reduced sperm motility, and induced sperm apoptosis [78]. Once initiated, LPO in spermatozoa results in the accumulation of lipid peroxides in the sperm plasma membrane. Supplementation of transition metal ions, such as Fe^2+^, accelerates LPO, which in turn leads to a loss of many sperm functions [79]. These peroxides can induce DNA damage and decrease the fertilizing ability of sperm. While associated with diminished sperm function and viability, lipid peroxides also enhance the ability of spermatozoa to bind with homologous and heterologous zona pellucida [80].

## 3. Laboratory Diagnostic Tests for OS-Associated Male Infertility

The concept that an imbalance in redox within the male reproductive tract or seminal secretions might impact fertility has been widely acknowledged to modulate human sperm function and conception outcomes [81]. The identification of markers for cellular OS allows for understanding disease pathobiology, prognosis, and treatment responses [81,82]. Tests measuring ROS levels, TAC, and biomarkers associated with oxidative damage quantify the extent of OS [82,83,84].

Sperm DNA damage is often measured as a consequence of OS, although several other research methods exist to directly examine the balance of antioxidants and ROS. These assays, however, should be cautiously used and interpreted in clinical diagnostics until more conclusive evidence of their diagnostic relevance is available. Despite this caveat, the assays described herein are widely employed in andrology research and in some clinical diagnostic and assisted reproduction laboratories. Various assays exist to evaluate redox balance or ROS, differing in both methods and the type of ROS they detect (Table 3).

### 3.1. Chemiluminescence Method

The luminol method relies on the chemiluminescent response of luminol when reacting with free radicals. This response is measured by luminometry, calculating the number of relative light units (RLUs) per 1 × 10^6^ sperm. However, luminometers are generally not accredited for in vitro diagnostics; they lack standardization in design and calibration, and they are sensitive to sample handling and timing of measurement. Additionally, the described variation between machines and methodologies, coupled with the low quality of studies for prognosis, results in a lack of agreed reference values. Furthermore, luminol’s sensitivity to pH, temperature changes, and interference from chemicals complicate its application [85]. 

Chemiluminescence assay stands as the predominant direct test utilized for the quantification of ROS in semen [86]. The luminometer quantifies chemiluminescent signals in RLUs, calculated by averaging RLU values from both test and control tubes. ROS sample results are derived by subtracting the average RLU of the negative control and normalizing for sperm concentration per mL. Agarwal et al. examined 258 infertile men and 92 controls, proposing a cut-off value of <102.2 RLU/s/10^6^ sperm/mL to distinguish between fertile and infertile men [87].

### 3.2. Total Antioxidant Capacity 

The TAC assessment evaluates the ejaculate’s ability to balance OS by measuring the TAC of seminal plasma [85]. Numerous techniques are available in the literature for evaluating seminal TAC, differing in principles, methodologies, and sensitivities to various antioxidants. Consequently, outcomes may vary depending on the chosen testing method [88]. The 2,2-Diphenyl-1-Picrylhydrazyl (DPPH) method utilizes spectrophotometric evaluation, relying on the color change of DPPH in the presence of antioxidants [89]. Trolox equivalent antioxidant capacity (TEAC) assesses antioxidant concentration spectrophotometrically using oxidized substrates [90]. An ELISA-based assay measures the formation of the 2,2′-Azinobis (3-ethylbenzothiazoline-6 sulfonic acid) radical, offering a high-throughput alternative to spectrophotometric detection [91]. The Oxygen Radical Absorbance Capacity assay quantifies antioxidants’ ability to inhibit substrate oxidation by exogenous oxidants using fluorescent probes. The Ferric Reducing Antioxidant Power test directly assesses TAC by monitoring the reduction of Fe^3+^ to Fe^2+^ by antioxidants [92]. Enhanced chemiluminescence assays evaluate antioxidant concentration indirectly through the reduction of chemiluminescent signals [93]. 

Each method has its advantages and limitations, requiring careful consideration in the interpretation of results. Similar to other assays, TAC measurement faces challenges such as a lack of evidence-based reference limits and variation between machines and methodologies [85]. Some of these tests have proposed cut-off values for different conditions or states. For example, in the DPPH method, a cut-off value of TAC levels was proposed to discriminate patients according to their fertility status [86]. Similarly, the TEAC method proposed a cut-off value to discriminate patients based on their oxidative seminal status. These cut-off values are used to classify individuals into different categories based on their antioxidant capacity levels [90]. 

### 3.3. Oxidation-Reduction Potential 

Another technique is the oxidation-reduction potential (ORP), also known as redox potential, which directly assesses the redox balance of a sample electrochemically. ORP quantifies electron transfer between chemical species [94]. In semen, ORP is assessed using MiOXSYS (Aytu BioScience, Inc., Denver, CO, USA), a galvanostat-based technology that gauges electron exchanges between antioxidants and oxidant species, offering insights into the present redox equilibrium. To conduct the test, the sample is placed into a designated sensor, which is then inserted into the device, initiating the test automatically [13]. Challenges include sample viscosity, poor liquefaction affecting sample flow, and the need for a standardized time of analysis post-ejaculation [85].

Initially, the MiOXSYS provides results in millivolts (mV). These values are then adjusted based on sperm concentration, with the final readings expressed as mV per 10^6^ sperm per mL. ORP values are affected by the concentration of viable cells in the ejaculate, thus fluctuating with sperm concentration. Additionally, the presence of morphologically immature or abnormal sperm with reduced motility impacts ROS production and the availability of antioxidants, leading to variations in ORP. Hence, two samples containing an equal number of spermatozoa but in different physiological states and OS levels will exhibit distinct ORP readings [14].

### 3.4. Nitroblue Tetrazolium 

The nitroblue tetrazolium (NBT) assay is a method used to determine cytoplasmic ROS levels by measuring the reduction of NBT to formazan crystals, which are easily detectable [95]. This reduction process is facilitated by the HMP pathway in sperm cytoplasm, generating superoxide anion [96,97]. This easy-to-perform, fast, and inexpensive assay has the advantage of detecting intracellular ROS, allowing for discrimination between different cellular sources in a heterogeneous cell population [96]. It can also detect low concentrations of neutrophils in the ejaculate [98]. However, its specificity in detecting ROS is questionable due to potential nonspecific signals generated by electron donors other than ROS. While the NBT assay has clinical significance in assessing seminal quality, sperm DNA damage, and OS, it is not routinely performed in andrology laboratories due to its lack of specificity and reference values. Larger multicenter trials are needed to further explore its application for OS testing. 

The test sample results are quantified as micrograms of formazan per 10^7^ cells and compared against a standard absorbance curve generated using known concentrations of the formazan substrate. Tunc et al. proposed a cut-off value of 24 micrograms of formazan per 10^7^ sperm to differentiate between fertile and infertile men [95]. Meanwhile, Amarasekara et al. reported cut-off values of 40.57 and 42.02 micrograms of formazan per 10^7^ sperm, which demonstrated high sensitivity and specificity in discriminating asthenozoospermic and unexplained infertile men from fertile men, respectively [99].

**Table 3:** Laboratory tests used to evaluate seminal OS.

Test Name	Approach	Advantages	Disadvantages	Cut-off Value
Chemiluminescence	Relies on chemiluminescent response of luminol with free radicals	- Detects ROS levels - Widely used in research - Relatively fast and inexpensive	- Not accredited for in vitro diagnostics - Lack of standardization - Sensitive to sample handling and timing - Lack of agreed reference values - Sensitivity to pH, temperature changes, and interference from chemicals	<102.2 RLU/s/10^6^ sperm/mL [87]
Oxidation-reduction Potential	Directly assesses redox balance electrochemically	- Promising method - Minimal sample manipulation - Standardizable	- Lack of strong evidence base - Sample viscosity affects analysis - Requires standardized analysis time	1.34 mV/10^6^ sperm/mL [14]
Total Antioxidant Capacity	Evaluates ejaculate’s ability to balance oxidative stress	- Measures overall antioxidant capacity - Uses Trolox standard for comparison - Can be used across various body fluids	- Lack of evidence-based reference limits - Variation between machines and methodologies	1947 μM trolox equivalent [90]
Nitroblue Tetrazolium	Measures cytoplasmic ROS levels by detecting reduction of nitroblue tetrazolium to formazan crystals	- Detects intracellular ROS - Discriminates between different cellular sources - Detects low neutrophil concentrations	- Questionable specificity for ROS detection - Lack of reference values - Not routinely performed	24 μg formazan/10^7^ [95] or 40.57 μg formazan/10^7^ [99]
Cytochrome C Reduction Test	Identifies extracellular superoxide anion by evaluating reduction of cytochrome c with NADPH-cytochrome c reductase	- Quantifies superoxide anion during respiratory burst - Effective for isolated enzyme analysis	- Limited in detecting small superoxide anion quantities - Cannot access intracellular space	NA
Electron Spin Resonance	Measures oxygen radicals using magnetic resonance spectroscopy	- Discriminates between various oxidative molecules - Provides spectral analysis of radicals	- Limited by potential unspecific adduct formation - Susceptible to antioxidant scavenging actions	NA
Flow Cytometry using DCFDA and DHE dyes	Profiles cells in heterogeneous fluid mixtures based on laser–cell interactions	- Fast, accurate, and reproducible - Enables multi-parametric analysis - Detects generalized radicals	- Requires cell-permeable reagents - Limited by lack of standardization in clinical utility	NA

ROS: reactive oxygen species; RLU: relative light unit; NA: not available; NADPH: nicotinamide adenine dinucleotide phosphate; DFDA: dichlorodihydrofluorescein diacetate; DHE: dihydroethidium.

### 3.5. Cytochrome C Reduction Test

The cytochrome c reduction test is a colorimetric assay designed to identify extracellular superoxide anions. It evaluates the reduction of cytochrome c by NADPH-cytochrome c reductase (NCR) in the presence of NADPH, with analysis conducted spectrophotometrically by measuring absorbance at 550 nm [100]. This test is effective for quantifying superoxide anions released during the respiratory burst of neutrophils or by isolated enzymes. To specifically assess the rate of superoxide anion-mediated reduction, the test incorporates the addition of superoxide dismutase (SOD) enzyme [100]. SOD facilitates the dismutation of superoxide anion into hydrogen peroxide, and the determination of a SOD-inhibitable signal is employed for result normalization. Results are typically expressed as NADPH-cytochrome c reductase (NCR) units; one unit of NCR activity corresponds to the enzyme generating 1 nanomole of cytochrome c reduction per minute. However, this test has limitations in detecting small quantities of superoxide anions and cannot access the intracellular space, thereby limiting the detection of the extracellular ROS fraction [101].

### 3.6. Electron Spin Resonance 

Electron Spin Resonance (ESR) or Electron Paramagnetic Resonance (EPR) is a technique utilized to measure oxygen radicals based on magnetic resonance spectroscopy [102]. EPR spectroscopy can also assess sperm membrane fluidity using lipophilic probes [103]. Electrons, characterized by a spin quantum number (ms), can have values of ±1/2. Under the Maxwell–Boltzmann distribution, there are typically more electrons in the lower energy state with ms equal to −1/2 [104]. When subjected to a fixed frequency of microwave irradiation, electrons are excited from the lower to the higher energy level (resonance), resulting in energy absorption, which is monitored and converted into a spectrum [104]. ESR measurements yield insights into the quantities, types, nature, surrounding environment, and behavior of unpaired electrons. 

Strategies like the “spin-trap” or hydroxylamine spin probes were developed to detect short-lived ROS [105]. The spin-trap approach involves diamagnetic compounds that trap radical molecules, generating detectable radical adducts via ESR. Hydroxylamine spin probes, on the other hand, are oxidized to stable nitroxide, which accumulates and can be detected by ESR. The “ideal” spin-trap molecule should be highly soluble, yield stable spin-adducts, be insensitive to inadvertent photolysis, and be specific for radicals. Although the spin-trap strategy enables discrimination between various oxidative molecules, it has limitations. Chemical modification by enzymes can lead to the formation of unspecific adducts regardless of oxidative molecule concentration. Additionally, antioxidant scavenging actions, such as those of SOD and ascorbate present in the sample, can hamper adduct formation [102]. 

### 3.7. Flow Cytometry-Based Intracellular ROS Measurement Using DCFDA and DHE Dyes

Flow cytometry, utilizing laser-based technology, counts, sorts, and profiles cells in heterogeneous fluid mixtures [101]. Laser–cell interactions are assessed by measuring light scatter and fluorescence intensity, using dyes or monoclonal antibodies that target specific extracellular or intracellular molecules. The signal detection allows for simultaneous multiparametric analysis of the physical and chemical characteristics of up to thousands of particles per second. Intracellular ROS detection employs cell-permeable reagents. Two specific dyes, dichlorodihydrofluorescein diacetate (DCFDA) and dihydroethidium (DHE), measure intracellular ROS. DCFDA, after cellular esterase de-acetylation, is oxidized by ROS into fluorescent DCF, emitting green fluorescence. DHE is oxidized by intracellular superoxide anions into ethidium bromide, emitting red fluorescence [101]. DCFDA measures peroxyl, alkoxyl, nitric oxide, carbonate, and hydroxyl radicals as well as peroxynitrite within the cell, while DHE specifically detects intracellular superoxide anions.

Flow cytometry is fast, accurate, reproducible, and sensitive to changes in the cellular redox state, enabling the tracking of ROS variations over time. Incubation with DCFDA and DHE probes allows for the measurement of generalized radicals, even in samples with low sperm counts [101]. The levels of ROS in assessed sperm cells are indicated as a percentage of fluorescence intensity [13]. For sample preparation, DCFDA (25 μM) and DHE (1.25 μM) are prepared in the dark to avoid the decay of the fluorescent signal. Samples are incubated with DCFDA or DHE at 37 °C for 40 min in the dark. A positive control treated with an organic peroxide, 500 μM tert-butyl hydrogen peroxide, is included in the analysis. Samples are incubated in propidium iodide (PI—1.25 μg/mL), a red-fluorescent nuclear and chromosome counterstain, to simultaneously identify dead cells. Flow cytometry analysis requires examining a minimum of 10,000 spermatozoa per assay. ROS levels in analyzed sperm cells are expressed as the percentage of fluorescence intensity.

Occasionally applied in advanced clinical practice for male infertility investigation, flow cytometry reveals OS in samples with low leukocytospermia [102]. Ghaleno et al. analyzed the intracellular ROS concentration by flow cytometry in semen selected by density gradient centrifugation as well as direct and conventional swim-up [106]. The increase of hydrogen peroxide associated with the application of conventional swim-up suggested that the washing and removal of semen plasma caused reduced antioxidant activity. A negative correlation was observed between seminal hydrogen peroxide and pronuclear formation [106]. While these assays provide valuable insights into the redox balance and ROS levels in semen, their clinical utility remains limited until further evidence establishes their diagnostic relevance and standardization. Continued research is crucial to understanding the role of flow cytometry methodologies in evaluating male fertility and reproductive outcomes [13].

## 4. Therapeutic Modalities to Reduce Seminal OS

**Figure 2:**
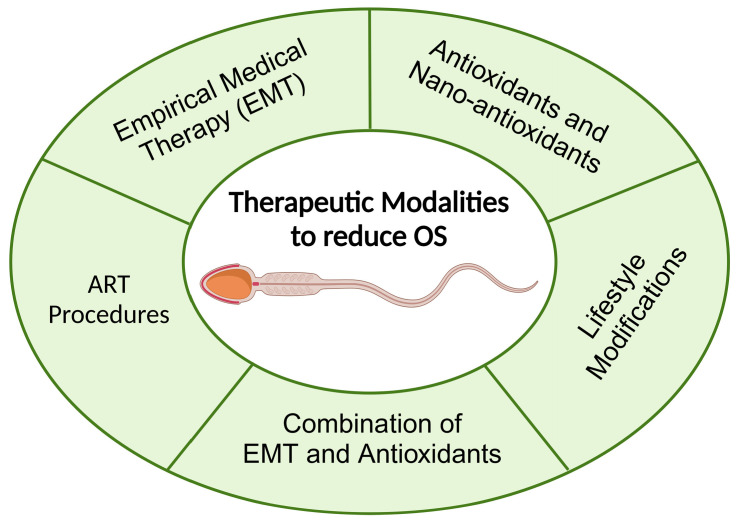
Therapeutic options available to reduce seminal OS. ART: artificial reproductive techniques; IVF: in vitro fertilization; ICSI: intracytoplasmic sperm injection.

Although several advancements have been made in assessing OS in semen, clear protocols for managing idiopathic and OS-related male infertility remain elusive [107]. This section explores the existing literature on treatment modalities (Figure 2), including empirical approaches for men with increased ROS levels, targeted therapies, and strategies for mitigating seminal OS. 

### 4.1. Empirical Medical Therapy

As established above, leukocytospermia is associated with increased OS, thus negatively impacting male fertility. A systematic review revealed that treating leukocytospermia with broad-spectrum antibiotics resulted in resolved leukocytospermia in five of eight clinical trials. Notably, frequent ejaculation at least every 3 days along with antibiotics was found to be more effective than antibiotics alone. Three clinical trials showed statistically significant improvement in sperm concentration and motility after treatment with antibiotics [108]. Another study demonstrated that treatment of low-level leukocytospermia with doxycycline therapy significantly increased the natural pregnancy rate relative to the controls [109]. 

Furthermore, hormone replacement therapy is commonly used to treat idiopathic male infertility. Due to the limitations of available diagnostic tools (i.e., not sensitive enough), hormonal profiles in these men may not be detectable on standard hormone panels. Many of them receive hormonal therapy involving gonadotropins, androgens, selective estrogen receptor modulators (SERMs), and aromatase inhibitors [110]. These drugs primarily aim to inhibit or reverse the activity of the hypothalamic–pituitary–gonadal axis to enhance spermatogenesis. Hormonal treatments have demonstrated significant efficacy, particularly in individuals with IHH [111]. While both SERMs and aromatase inhibitors hold promise for off-label use in the treatment of idiopathic male infertility, further large-scale randomized controlled trials incorporating data on pregnancy outcomes are needed to provide more conclusive evidence.

### 4.2. Antioxidant Treatment

Antioxidants are classified into enzymatic and nonenzymatic types based on their activity and chemical structure [112], and they occur in both intracellular and extracellular environments [113]. Enzymatic antioxidants, with the contribution of some trace elements such as zinc, iron, magnesium, and copper, convert ROS into hydrogen peroxide and ultimately into water [113]. Nonenzymatic antioxidants, such as vitamin C, vitamin E, melatonin, curcumin, and reduced glutathione, neutralize free radicals by abolishing their chain reactions [114]. Clinical trials have determined the effect of antioxidant supplementation on seminal fluid OS as well as on sperm parameters. One study evaluating OAT men before and after antioxidant supplementation demonstrated improvement in sperm count, progressive motility, viability, and morphology, along with reduced levels of OS. The percentage of fertilization and embryos obtained was also increased [115]. in another study, the supplementation of Coenzyme Q10 (CoQ10) in infertile men for 3 months resulted in significantly improved sperm concentration, progressive and total motility, as well as TAC, along with reduced levels of ROS [116]. Systematic reviews and meta-analyses have demonstrated that antioxidant therapy, such as vitamins E and C, carnitines, N-acetyl cysteine, CoQ10, zinc, selenium, and folic acid, leads to improved sperm parameters [117]. A Cochrane database systematic review demonstrated that antioxidant therapy can increase clinical pregnancy rates and birth rates [26]. Supplementation with both vitamins C and E has been shown to enhance semen oxidative stability, especially when vitamin E levels are adequate. Conversely, decreased levels of vitamin E have been associated with reduced oxidative stability. Therefore, combined therapy with vitamin E and vitamin C appears to enhance the viability and motility of spermatozoa, ultimately leading to increased fertility rates [118]. However, caution should be exercised against excessive use of antioxidants such as vitamin C, vitamin E, and N-acetyl cysteine, as it can lead to reductive stress; this phenomenon can be as harmful as OS [119]. 

A combination of empirical medical therapy along with antioxidant therapy may also be considered to enhance pregnancy rates. A study evaluating combined antibiotic and antioxidant treatment observed a reduction in leukocytes in semen, accompanied by significantly increased antioxidant levels and decreased NO levels [120]. Combinations of different hormonal therapies, such as tamoxifen with an androgen-like testosterone undecanoate, have demonstrated significant improvements in sperm parameters and spontaneous pregnancy rates among men with iOAT and normozoospermic men with female factor subfertility [121,122]. Other small, randomized trials have explored combinations of hormonal therapy with antioxidants, such as clomiphene with L-carnitine or vitamin E, which also revealed positive effects on sperm parameters and pregnancy rates [123,124]. Moreover, a recent meta-analysis indicated a positive impact of antioxidant combinations on pregnancy rates [26,125]. While some discrepancies exist among these studies regarding the extent of improvement in sperm parameters, treatment regimens combining zinc, vitamins, selenium, and N-acetyl cysteine have consistently demonstrated enhancements in sperm motility and/or concentration [41,126,127,128,129].

### 4.3. Nanoantioxidants in the Treatment of Seminal OS

Male reproduction and infertility treatments have benefited from advancements in nanotechnology [130]. As a novel approach with proven drug delivery, high bioavailability, and lower toxicity, nanoparticles (NPs) have been extensively studied for their potential applications in diagnosing and treating male infertility [131]. While antioxidants play a vital role in protecting cells from OS damage and have exhibited promising effects in treating male infertility, they possess certain drawbacks, including low solubility and low stability, which can result in fluctuating bioavailability [132]. 

The use of nanoantioxidant formulations resulted in increased durability, cellular uptake, and targeted delivery compared to antioxidants [112]. Different methods are available to produce nanoantioxidants, including supercritical fluid technology, emulsion/solvent evaporation, solvent displacement, templating, and the nanoprecipitation technique [133]. Animal studies reporting the beneficial effects of nanoantioxidants on the male reproductive system are summarized in Table 4. An evaluation of the oral administration of zinc oxide NPs in diabetic male rats demonstrated a protective effect against diabetes-induced OS due to the antioxidant and anti-inflammatory properties of zinc oxide, with lower toxicity levels [134]. The inclusion of zinc oxide NPs (30 mg/kg/diet) in the diet of rabbit bucks under severe heat stress resulted in improved sperm viability and concentration, increased testosterone levels, reduced cortisol levels, and enhanced serum seminal antioxidant activity [135]. Zinc NPs have shown a protective effect on cryopreserved sperm and could reduce apoptosis and improve sperm quality [136].

Due to their antioxidant auto-regenerative potential and low toxicity, cerium dioxide NPs have shown promising therapeutic results [137,138]. Recent research has shown that cerium dioxide NPs can scavenge hydroxyl or nitric oxide radicals and exhibit superoxide dismutase (SOD) and catalase mimetic activity [139,140,141]. Recent studies confirmed that cerium NPs enhance total thiol and antioxidant capacity [142,143]. Male rats treated with a single oral dosage of 1 mg/kg of cerium dioxide NPs (2–5 nm) showed an increased rate of spermatogenesis [144]. Sperm parameters such as count, motility, and viability were significantly improved in rats co-treated with cerium NPs [145]. A 10-day course of cerium dioxide NPs resulted in elevated sperm count, enhanced quantitative sperm parameters, significantly reduced serum LPO product levels, and increased catalase and SOD activity [146]. These antioxidative capabilities of cerium dioxide NPs have potential in the treatment of OS-associated male infertility [146].

Apart from zinc and cerium, other nanoantioxidant formulations, such as curcumin, selenium, and vitamin E, were reported to reduce OS and improve sperm function or parameters [147]. Varicocele-induced rats orally treated with nano-curcumin showed improvement in testicular function with decreased ROS and LPO levels and reduced sperm DNA fragmentation [148]. The injection of iron oxide NPs coated with curcumin in a group of mice (exposed to testicular hyperthermia) resulted in higher testosterone levels, lower apoptotic spermatozoa, and increased germ cell proliferation compared to other groups [149]. In another study, nano-emulsion of vitamin E exhibited a protective effect in cryopreserved sperm mixtures by reducing ROS, LPO, and damaged DNA. Moreover, it improved sperm viability and maintained spermatozoa mitochondrial activity [150]. Hozyen et al. demonstrated that the oral administration of selenium NPs (0.5 mg/kg bwt) in deltamethrin-induced infertile male rats improved sperm count, motility, and viability, while also enhancing antioxidant levels, glutathione peroxidase activity, and testosterone levels [151]. Similarly, several studies have demonstrated the ameliorative effect of selenium NPs on sperm parameters by reducing OS damage [152,153,154,155,156]. 

In the literature, studies evaluating the impact of nanoantioxidants on semen parameters involving human subjects are very scarce. A randomized clinical trial assessing curcumin nanomicelles in the treatment of male infertility revealed that nanocurcumin supplementation significantly enhanced sperm parameters, including total sperm count, sperm concentration, and motility [147].

**Table 4:** Selected animal studies reporting the beneficial effects of nanoantioxidants on the male reproductive system.

Study Reference	Nano Antioxidant	Purpose/Objective	Findings/Results
Sadraei et al. [148]	Curcumin nanoparticles	To evaluate the effect of curcumin nanoparticles on varicocele-induced rats’ reproductive parameters	Reduced ROS levels, decreased lipid peroxidation, lower sperm DNA fragmentation, and improved testicular function
El-Gindy et al. [135]	Nano-zinc oxide	To assess the impact of nano-zinc oxide supplementation on heat-stressed rabbit bucks’ sperm parameters	Increased sperm viability and concentration, elevated testosterone levels, reduced cortisol levels, and enhanced seminal antioxidant capacity
Jurado-Campos et al. [150]	Vitamin E nanoemulsion	To investigate the protective effect of vitamin E nanoemulsion on cryopreserved sperm mixtures	Reduced ROS levels, decreased lipid peroxidation, improved sperm viability, and maintained mitochondrial activity
Hozyen et al. [151]	Selenium nanoparticles	To examine the therapeutic effect of selenium nanoparticles on deltamethrin-induced infertility in male rats	Improved sperm count, motility, viability, and antioxidant levels; enhanced testosterone levels
Khalil et al. [156]	Selenium and zinc nanoparticles	To evaluate the protective effect of selenium and zinc nanoparticles on cryopreserved spermatozoa mixtures	Reduced apoptosis, improved sperm quality, and protection against oxidative stress
Alizadeh et al. [147]	Curcumin nano-micelles	To investigate the efficacy of curcumin nano-micelles in treating male infertility via a randomized clinical trial	Significantly improved sperm parameters, including count, concentration, motility, and morphology
Jurado-Campos et al. [130]	Vitamin E nanoemulsion	To assess the use of nano controlled-release antioxidants in preventing oxidative stress in an ART setting	Support for using nano controlled-release antioxidants to prevent OS in ART
El-Behery et al. [134]	Zinc oxide nanoparticles	To evaluate the protective effect against diabetes-induced oxidative stress in diabetic male rats	Protective effect against diabetes-induced oxidative stress with lower toxicity
Afshar et al. [149]	Iron oxide nanoparticles coated with curcumin	To investigate the effect on testosterone levels, apoptotic spermatozoa, and germ cell proliferation in mice exposed to testicular hyperthermia	Increased testosterone levels, lower apoptotic spermatozoa, and more germ cell proliferation relative to other groups

### 4.4. Lifestyle Modifications in the Management of Seminal OS

Addressing diet and lifestyle factors implicated in OS can potentially improve male fertility. Nutritional components of a diet can contribute positively or negatively to infertility. Diets rich in fruits, vegetables, fish, low-fat dairy products, and macronutrients, including omega-3 fatty acids and antioxidants, have been associated with improved sperm parameters and better semen quality [157]. Unsaturated fats are recommended over saturated fats and can be obtained from sources such as olive oil, oily sea fish, nuts, and seeds. Diets low in saturated and trans fats were associated with improved sperm parameters. Adherence to a Mediterranean or similar dietary pattern, which is rich in fruits, vegetables, fiber, seafood, nuts, seeds, and vegetable oils, as well as antioxidant-rich plant-based foods, can encompass many of the above-listed recommendations and is associated with improved semen quality [158,159,160,161]. However, high-sugar diets with processed meats, refined flour, soy foods, and full-fat dairy products are associated with diminished sperm parameters, and intake of such products should be reduced [157]. 

Weight loss, adiposity, and exercise are other lifestyle facets that can factor into male infertility. Weight loss has been shown to improve endocrine parameters, hormone regulations involved in the hypothalamic–pituitary–gonadal axis, DNA fragmentation, as well as increase sperm retrieval rates on micro-testicular sperm extraction [4]. Clinical evidence suggests that reducing adiposity through diet and exercise can lead to improvements in semen parameters, even without a reduction in BMI [162,163,164]. Weight loss in obese fathers can enhance embryo quality, development, and metabolic function in offspring [165]. Moderate regular exercise can also improve fertility parameters and reduce OS and DNA fragmentation [166]. Recent studies have reported that the integration of regular yoga practices can decrease seminal OS as well as improve sperm parameters and DNA integrity [167,168] 

Lifestyle modifications, such as quitting tobacco, are crucial for improving altered sperm parameters [169]. Smoking cessation has been linked to decreased OS and improved semen parameters [170,171]. Limiting alcohol consumption (<5 units per week) may be safe, but moderate and heavy alcohol consumption (>25 units per week) should be avoided [172,173,174]. Similarly, substance use, including cannabis and opiates, has been shown to decrease sperm parameters and should be avoided [175].

Mental health and psychological stress are also recognized as contributors to the infertility picture. Psychological stress management through mind–body practices, meditation, and yoga has been shown to improve male fecundity and decrease OS [176,177]. While further research is needed to explore the effects of stress-release techniques and therapeutic approaches, such as cognitive behavioral therapy and mindfulness, these methods can assist in stress management while also assisting in managing psychologic stress related to sexual performance and fertility outcomes [176,178]. Adequate sleep is another essential factor that may improve semen quality [179,180]. It is important to note that specific thresholds for many lifestyle parameters remain undetermined and require further investigation.

### 4.5. OS-Associated Male Infertility and Assisted Reproductive Techniques 

Management of male factor infertility involves assisted reproductive techniques (ARTs), which include intrauterine insemination (IUI), in vitro fertilization (IVF), and intracytoplasmic sperm injection (ICSI). IVF and ICSI have brought about a revolutionary shift in the treatment of male factor infertility. While IVF was initially considered a potential solution for male infertility, its effectiveness was limited by poor fertilization rates in cases of impaired semen parameters [181]. However, the advent of ICSI in 1992 marked a significant breakthrough in the management of severe male factor infertility [182]. ICSI involves the precise injection of a single spermatozoon directly into the cytoplasm of an oocyte, typically obtained from follicles following controlled ovarian hyperstimulation.

Generally, IUI is a first-line therapy in a couple where the female partner has normal fertility status and at least 5 × 10^6^ total motile spermatozoa. If pregnancy is not achieved after three to six cycles of IUI, IVF can be conducted as the next step in treatment [183]. IVF, which involves the fertilization of eggs and sperm in a laboratory setting outside of the body, can be conducted if sperm counts are less than 20 × 10^6^ but there is reasonable motility present [9]. ICSI is suggested when less than 0.5 × 10^6^ motile spermatozoa are obtained [183].

Concerns regarding fertilization failure have prompted an increased reliance on ICSI. OS is correlated with poor IVF outcomes. Redox balance of the ART media and regents plays an important role in the development of the embryo. Maldonado Rosas et al. reported increased blastocyst formation and pregnancy rates by adjusting the redox state of the culture media similar to that of follicular fluid from oocyte donors using a combination of antioxidants [184]. Furthermore, seminal OS levels were able to predict the fertilization and blastulation rates in patients undergoing ICSI [185,186]. However, these pilot reports need to be evaluated in large cohorts, and more evidence is required to fully understand the role of OS in ART practice.

Lifestyle modifications and antioxidant treatment have been recommended to decrease sperm DNA fragmentation [187]. It has been proposed that testicular sperm extraction can be utilized to select healthy sperm with good chromatin integrity. A recent meta-analysis concluded that testicular sperm exhibit lower levels of sperm DNA fragmentation than ejaculated sperm. It has been suggested that infertile couples may benefit from the use of testicular sperm for ICSI if the male partner has a high sperm DNA fragmentation rate in the ejaculated sperm [188]. Studies have reported a higher percentage of protamine-deficient and DNA-fragmented spermatozoa in infertile men with globozoospermia compared to fertile men. Consequently, it has been suggested that increased sperm DNA damage in globozoospermia is likely related to defective DNA compaction. Antioxidant therapy before ICSI and artificial oocyte activation could be a potential strategy to address this issue [189].

## 5. Conclusions

Many factors, both on a macroscopic and microscopic level, contribute to male infertility. ROS have a physiological role throughout all stages of fertilization, which is important to consider. Furthermore, it is essential to consider the many factors that can increase ROS that negatively impact fertility, including many endogenous and exogenous agents. Male infertility necessitates a multifaceted approach that integrates both diagnostic and therapeutic interventions. Diagnosis to determine if ROS are involved in male infertility is essential, with multiple laboratory tests available to identify this, including seminal ORP assay. Empirical medical therapy, coupled with antioxidant supplementation and lifestyle modifications, offers the potential to decrease OS and enhance sperm parameters. However, the efficacy of these interventions remains to be fully elucidated, especially within the context of personalized treatment plans tailored to individual patient profiles. By integrating innovative diagnostic techniques with evidence-based therapeutic modalities, clinicians can tailor treatment plans to address the specific needs of each patient, thereby optimizing the management of OS-associated male infertility and increasing the likelihood of successful conception for couples.

## Figures and Tables

**Table 1:** Proteomic studies analyzing the impact of OS on sperm proteins.

Study Reference	Subjects	Key DEPs	Findings
Hamada et al. [68]	20 donors and 32 infertile men’s semen samples, categorized into ROS +ve and ROS −ve groups	lactotransferrin-2, peroxiredoxin-1	Identification of 1265 and 1343 proteins in ROS +ve and ROS −ve groups, respectively, highlighting potential markers for oxidative stress in sperm
Sharma et al. [69]	Investigation involving 20 normal donors and 32 infertile men’s semen samples, segregated by ROS status	HIST1H2BA, MDH2, TGM4, GPX4, GLUL, HSP90B1, HSPA5	Identification of 74 DEPs, suggesting alterations in energy metabolism, gluconeogenesis, glycolysis, and oxidative stress regulation in ROS+ group
Ayaz et al. [70]	Analysis of 42 semen samples from infertile men (low, medium, and high ROS groups) and 17 fertile men (control)	CLGN, TPPII, DNAI2, EEA1, HSPA4L, SERPINA5	Detection of 305 DEPs, with 51 unique to low ROS, 47 to medium ROS, and 104 to high ROS groups, indicating ROS-related protein expression changes

ROS: reactive oxygen species; DEPs: differentially expressed proteins; +ve: positive; −ve: negative.

**Table 2:** Proteomic studies analyzing the impact of OS on seminal plasma proteins.

Study Reference	Subjects	Key DEPs	Findings
Dias et al. [71]	Study involving 20 fertile men’s semen samples, stratified by ROS status	NDUFS1, SOD1, PRDX4	Identification of 371 DEPs in sperm and 44 DEPs in seminal plasma, indicating activation of antioxidant defense and proteasomal systems
Sharma et al. [72]	Examination of 20 donors and 32 infertile men’s seminal plasma samples, categorized by ROS status	FN1, PIP, KLK3, SEMG2, CLU, MIF, LTF	Detection of 14 DEPs associated with antioxidative activity and regulatory processes, providing insights into seminal plasma composition under ROS conditions
Agarwal et al. [73]	Analysis of 42 semen samples from infertile men (low, medium, and high ROS groups) and 17 fertile men (control)	MME, FAM3D	Overexpression of proteins linked to post-translational modifications, protein folding, and developmental disorders observed in the high ROS group
Intasqui et al. [74]	Investigation involving 46 normozoospermic samples categorized by lipid peroxidation levels	Mucin-5B	Identification of 94 DEPs and enriched pathways related to lipid metabolism, antioxidant activity, heat stress response, and immune regulation
Herwig et al. [75]	11 infertile iOAT men and 11 fertile men (control)	Panel of 46 proteins indicative of infertility	Identification of 2489 proteins and 5 proteins common between infertile OAT and fertile group

ROS: reactive oxygen species; iOAT: idiopathic oligoasthenoteratozoospermic; DEPs: differentially expressed proteins; OAT: oligoasthenoteratozoospermic.

## Data Availability

Not applicable.
